# Clinical features and treatment outcomes of intraocular and ocular adnexal metastasis

**DOI:** 10.1038/s41598-024-64464-z

**Published:** 2024-07-03

**Authors:** Wantanee Dangboon Tsutsumi, Apinya Rattanasuwan, Orapan Aryasit

**Affiliations:** Department of Ophthalmology, Faculty of Medicine, Prince of Songkla University, 15, Kanjanavanich Road, Kohong, Hat Yai, Songkhla, 90110 Thailand

**Keywords:** Lung cancer, Breast cancer, Intraocular metastasis, Ocular adnexal metastasis, Ocular adnexa, Overall survival, Medical research, Oncology

## Abstract

The aim of this study was to investigate the primary sites, clinical characteristics, and treatment outcomes of patients with metastatic tumors in the eye and ocular adnexa. This retrospective case series consisted of 42 patients diagnosed with intraocular metastasis (IM) or ocular adnexal metastasis (OAM) at a tertiary center between January 2001 and June 2023. The patients comprised 18 men and 24 women; 24 (57%) and 18 (43%) patients were diagnosed with IM and OAM, respectively. In the IM group, the primary tumors originated from the lungs (79%), followed by the breasts (17%). In the OAM group, the primary tumors originated from the breasts (33%). Previously, 57% of the patients had been diagnosed with cancer. In the IM group, 38% exhibited bilateral involvement. Only 6% of the patients with OAM had bilateral diseases. The 1-, 3-, and 5-year overall survival (OS) was 42%, 18%, and 7%, respectively. The median OS since metastasis diagnosis in the lungs and breast was 11.8 and 10.5 months, respectively. Lung cancer remains the predominant primary cancer in IM, whereas breast cancer is the major cancer in OAM. Despite poor OS, early detection will facilitate the prompt treatment of primary cancer and metastatic sites.

## Introduction

The choroid is most common site of metastatic tumors in the ocular region^1,2^. Metastatic tumors concerning the eye and ocular adnexa are considered the advanced stages of primary cancer^3^. Ophthalmologists unfamiliar with this condition find the diagnosis challenging because of numerous clinical manifestations. The incidence of cancer is increasing worldwide; therefore, the accurate diagnosis of metastatic tumors will help patients access appropriate treatments that will improve their survival. Furthermore, it will elucidate non-response to treatment as well as recurrence after cancer remission.

Several studies have reported on metastatic tumors of the eye and ocular adnexa in Western and Asian populations^4–8^. Shield et al.^1^ mentioned that uveal metastases in 1,111 patients mostly originated from the breast (37%) and lungs (26%); primary cancer was detected before uveal metastasis in 67% of the patients. Lee et al. from Korea reported data from 14 patients (15 eyes) with known primary cancers before intraocular metastasis (IM)^7^. These tumors originated in the breast (29%), lungs (29%), and stomach (29%).

Breast and lung cancers are the predominant tumors that metastasize to the uvea and orbit^4,8,9^. Approximately 12% of the patients present with ocular manifestations before the diagnosis of primary cancer^9^. 

The clinical characteristics and treatment outcomes of IM and ocular adnexal metastasis (OAM) have not been reported in large cohort studies conducted in Asian countries. This study aimed to identify the clinical features, visual prognosis, and survival outcomes of IM and OAM after cancer diagnosis in the Thai population. The findings will enable ophthalmologists to better understand these rare but deadly diseases and provide essential knowledge for establishing treatment protocols.

## Methods

The inclusion criterion was as follows: patients diagnosed with IM or OAM from solid tumors. Retina specialists, ocular oncologists, or oculoplastic specialists at the Songklanagarind Hospital confirmed the final diagnosis from January 2001 to June 2023. Patients diagnosed with hematologic malignancy and tumor invasion from adjacent structures were excluded. This retrospective study was approved by the Human Research Ethics Committee of the Faculty of Medicine, Prince of Songkla University (REC number 64-140-2-4).

Demographic data, fundus photographs, optical coherence tomography (OCT), ocular ultrasonography, orbital imaging, and tissue diagnoses were reviewed. Survival data, including the date and cause of death, were gathered from the Songkhla Cancer Registry and the Bureau of Registration Administration. Overall survival (OS) was defined as the period from the date of IM or OAM diagnosis until the date of death caused by any reason. Patients who survived were censored at the last known follow-up or November 02, 2023.

Data analyses were performed using the R program version 4.1.0 (The R Group, Vienna, Austria) with Epicalc software. Continuous variables are presented as mean, standard deviation, median, and interquartile range. Discrete variables are presented as percentages. Survival rates were calculated using Kaplan–Meier curves, and categorical variables were analyzed using the log-rank test. *P*-values < 0.05 indicated statistical significance.

### Ethics approval

The requirement for informed consent was waived by the Institutional Review Board of the Faculty of Medicine, Prince of Songkla University. All methods were performed in accordance with the relevant guidelines and regulations.

## Results

Twenty-four (15 unilateral and 9 bilateral) and 18 (17 unilateral and one bilateral) patients met the inclusion criteria for IM and OAM, respectively. The mean age was 49.9 years (range, 10 months to 78.1 years). The patients were diagnosed with IM based on clinical findings. Of the 18 patients, 14 were diagnosed with OAM based on tissue biopsy. Table 1 summarizes the demographic data and clinical features. IM from lung cancer was reported in 12 women and 7 men (Table 2).

**Table 1:** Clinical features of intraocular and ocular adnexal metastasis.

Clinical features	Total (*N* = 42, eyes = 52)	Type of metastasis	
		Intraocular metastasis (*N* = 24, eyes = 33)	Ocular adnexal metastasis (*N* = 18, eyes = 19)
Age of diagnosis (years)			
Mean ± SD	49.91 ± 20.97	53.35 ± 13.62	45.33 ± 27.77
Median, [IQR]	52.05 (44.43, 64.68)	52.05 (45.12, 64.06)	54.54 (24.80, 67.78)
Min, Max	0.87, 78.07	24.69, 75.64	0.87, 78.07
Sex			
Male	18 (42.9)	7 (29.2)	11 (61.1)
Female	24 (57.1)	17 (70.8)	7 (38.9)
First diagnosis location			
Eye	18 (42.9)	12 (50.0)	6 (33.3)
Body	24 (57.1)	12 (50.0)	12 (66.7)
Diagnosis			
Histopathologic report	17 (40.5)	3 (12.5)	14 (77.8)
Clinical feature	25 (59.5)	21 (87.5)	4 (22.2)
Primary tumor origin			
Lung	19 (45.2)	19 (79.2)	0 (0.0)
Breast	10 (23.8)	4 (16.7)	6 (33.3)
Other**s**	13 (31.0)	1 (4.2)	12 (66.7)
Laterality			
Unilateral	32 (76.2)	15 (62.5)	17 (94.4)
Bilateral	10 (23.8)	9 (37.5)	1 (5.6)
Blurred vision			
Yes	37 (71.1)	31 (93.9)	6 (31.6)
No	15 (28.9)	2 (6.1)	13 (68.4)
Diplopia			
Yes	7 (13.5)	0 (0.0)	7 (36.8)
No	45 (86.5)	33 (100.0)	12 (63.2)
VA at initial diagnosis			
Mean ± SD	0.89 ± 0.87	0.94 ± 0.76	0.78 ± 1.07
Median, [IQR])	0.50 (0.1, 1.6)	0.70 (0.3, 1.6)	0.25 (0.1, 1.1)
IOP at initial diagnosis			
Mean ± SD	13.53 ± 4.04	12.40 ± 3.18	16.82 ± 4.60
Median, [IQR])	13.0 (11, 15)	11.7 (10, 15)	15.0 (14, 22)

*N* Number of patients, *SD* Standard deviation, *IQR* Interquartile range, *VA* Visual acuity; and *IOP* Intraocular pressure.

**Table 2:** Sex and primary tumor origin.

Sex	Type of metastasis					
	Intraocular metastasis			Ocular adnexal metastasis		
	Lung (*N* = 19)	Breast (*N* = 4)	Others (*N* = 1)	Lung (*N* = 0)	Breast (*N* = 6)	Others (*N* = 12)
Male	7	0	0	0	0	11*
Female	12	4	1^&^	0	6	1^$^

*4 neuroblastoma, 3 prostate gland; 1 uterus, 1 rectum, 1 thyroid gland, and 1 melanoma of skin; ^&^ 1 uterus; ^$^ 1 liver.

Previously, 57% of the patients had been diagnosed with cancer. Lung cancer (74%) had a higher frequency of metastasis than breast cancer (20%) before primary tumor diagnosis (*P* = 0.006). The lung cancer histology was predominantly adenocarcinoma (84%).

**Figure 1:**
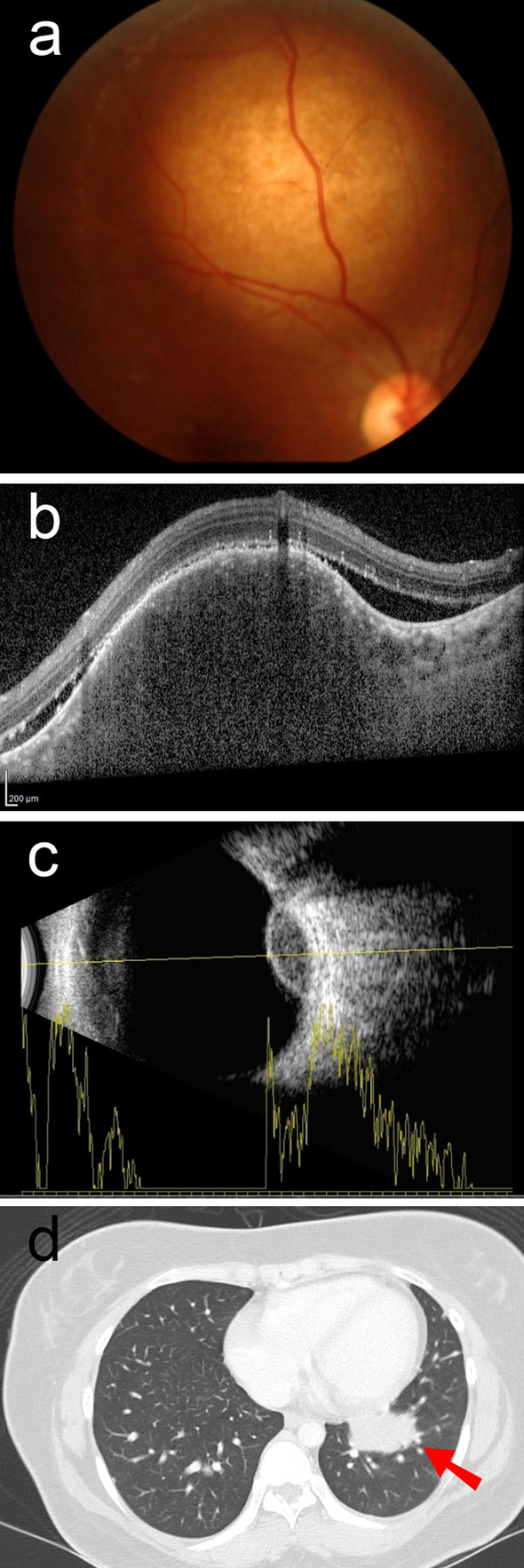
A 26-year-old woman presents with flashing and reduced vision in the right eye: (**a**) Color fundus photography indicates pale yellow choroidal mass; (**b**) Hypo-reflective elevated choroidal mass with subretinal fluid on optical coherence tomography; (**c**) Ultrasonography shows a hypoechoic dome shape mass with moderate variable amplitude of internal reflectivity on A-scan; and (**d**) Lung mass with poorly differentiated adenocarcinoma.

Among the patients with IM, the lungs (79%) were the most common origin of cancer; all patients had non-squamous cell carcinoma. Blurred vision (94%) was the most common symptom, followed by flashing (13%) and floaters (6%). All IMs were found in the choroid and were primarily located in the midperipheral fundus (55%). Single lesions per eye (77%), pale yellow color (71%), and dome-shaped configuration (50%) were the common characteristics of choroidal metastases (Fig. 1). For the OAM group, the most common clinical presentations were proptosis (58%), diplopia (37%), and blurred vision (32%).

Thirty patients underwent treatment. Table 3 summarizes the treatment modalities for IM and OAM. The mean visual acuity (VA) in Log MAR of the treatment group at 1, 3, 6, 12, and 24 months was 1.00, 0.65, 0.55, 0.58, and 0.10, respectively. For the observation group, the mean Log MAR VA at 1 and 3 months was 0.61 and 1.50, respectively. The comparison of VA between the treatment and observation groups at 1 and 3 months was statistically insignificant (*P* = 0.492 and *P* = 0.188, respectively).

**Table 3:** Treatment modalities for patients with intraocular and ocular adnexal metastasis.

Treatment modalities and primary tumor origin	Total (*N* = 30)	Type of metastasis	
		Intraocular metastasis (*N* = 15)	Ocular adnexal metastasis (*N* = 15)
Systemic chemotherapy			
Lung	3	3	0
Brest	6	2	4
Others*	6	0	6
Radiation			
Lung	2	2	0
Breast	2	0	2
Tyrosine kinase inhibitor			
Lung	4	4	0
Combined chemotherapy with radiation			
Lung	3	3	0
Liver	1	0	1
Combined chemotherapy with hormonal therapy			
Breast	1	1	0
High dose radioiodine therapy			
Thyroid gland	1	0	1
Radical prostatectomy			
Prostate gland	1	0	1

*4 neuroblastoma, 1 prostate gland, and 1 melanoma of skin.

**Figure 2:**
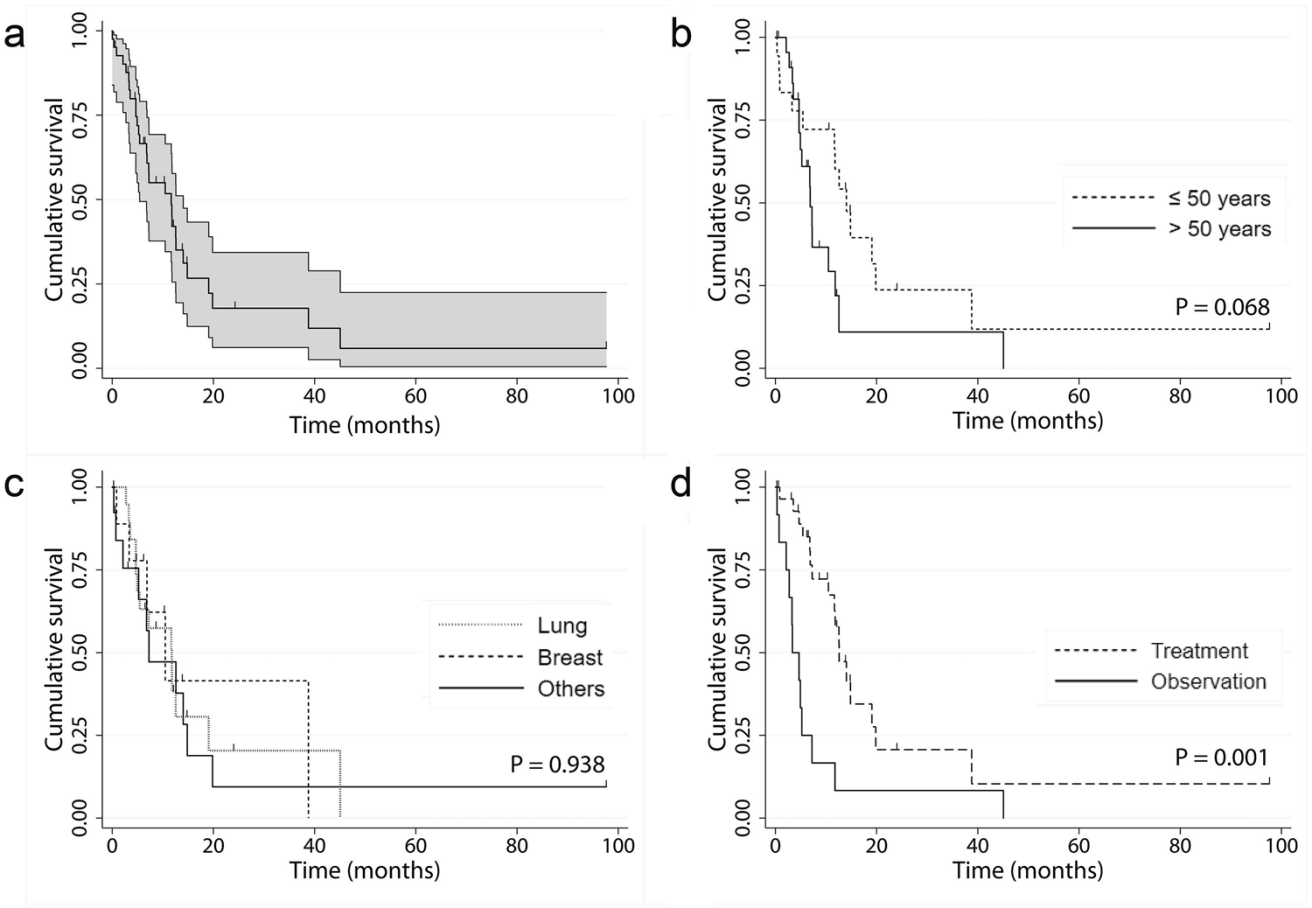
Kaplan–Meier survival estimates of the overall survival. (**a**) All patients; (**b**) age ≤ 50 years vs. > 50 years; (**c**) primary cancer origin; and (**d**) treatment group vs. observation group.

The mean follow-up duration was 11.7 months (range, 8 h to 8.1 years). Thirteen patients survived. The Kaplan–Meier survival estimates for OS at 1, 3, and 5 years were 42%, 18%, and 7%, respectively. The log-rank test identified the variables that were significantly associated with OS (Fig. 2). The factor affecting worse OS was the observation group (*p* = 0.001).

## Discussion

This retrospective study reports on the clinical features and treatment outcomes, including VA and OS, in 42 patients (52 eyes) with IM and OAM. The lungs and breasts were the most common cancer origins for IM. Breast cancer was the most common primary neoplasm in OAM, followed by neuroblastomas. The OAM group comprised various primary cancer origins. One-third of the patients with IM had bilateral involvement, and only 6% of the patients with OAM had bilateral disease. Blurred vision was a common symptom of IM, whereas proptosis, diplopia, and high IOP were more frequent symptoms in OAM. The 5-year OS of all the patients was poor (7%), particularly in the observation group.

The oculi and ocular adnexa are the rare regions of metastatic cancer. The uvea (63%) was the common site of metastasis, followed by the orbit (26%)^5^. The choroid and orbit were the most favored target sites for metastases development because of rich vascularization, compared with other locations. IM is reported in approximately 2% to 7% of the patients with cancer^10,11^. Orbital metastasis occurs in 3% to 4% of all orbital tumors^12,13^. This novel study compared the primary site, clinical characteristics, and survival between IM and OAM in Asians. The results of this study are consistent with previous findings; the common clinical features of intraocular metastasis include decreased VA, unilateral involvement, yellow discoloration, single-focal lesions, and choroidal location^1,2,14^. The mean age was 49.9 years (range, 10 months to 78.1 years), which is less than that reported in Shields' study (mean age 60 years)^1^, reflecting the variety in histology including neuroblastoma in young children and adenocarcinoma of the lungs in young adults.

The incidence of metastatic tumors in the eye and ocular adnexa has been increasing because of advanced diagnostic procedures, increased median survival of the patients with cancer, and the awareness of subspecialties for metastasis surveillance^15,16^. However, the actual numbers of IM and OAM were possibly higher than those reported in our study. The underestimation of actual incidence can be attributed to several factors. Patients with small lesions may remain asymptomatic or have undetectable lesions, particularly those with end-stage cancers^10^.

However, approximately 50% of the patients had no history of primary cancer before IM or OAM. The lungs were the most common primary tumor origin of IM in both sexes, inconsistent with previous reports^2,17^. In southern Thailand, lung cancer is the most common primary cancer in men, while cervical and breast cancers are the most common primary cancers in women^18^. The incidence of adenocarcinoma of the lung is expected to increase until 2030^19,20^, with a similar trend observed in breast cancer^21,22^. In contrast, in the United States, the most common cancers are breast, melanoma, and lung^15^. Consequently, the likelihood of metastatic tumors from lung cancer has increased in Thailand. A higher proportion of metastases from lung cancer was detected before the primary tumor than that from breast cancer. Thailand's national health system covers screenings for cervical, breast, and colorectal cancers^23^, but inadequate lung cancer screening programs and education may delay the detection of primary cancers. This study demonstrated that lung cancer was the most common primary cancer origin in females, differing from patterns in the United States due to the aggressive behavior of lung cancer and the earlier detection of breast cancer^1^.

In this study, three patients underwent enucleation because of suspected choroidal melanoma based on the clinical features and investigations; however, their histopathological diagnosis confirmed a metastatic tumor. Ophthalmologists should consider choroidal melanoma and perform extensive investigations, including ultrasonography, fluorescein angiography, indocyanine green angiography, and OCT, to exclude metastasis. This is because choroidal melanoma has a low incidence among Asians^24^. Additionally, positron emission tomography was used to determine the primary cancer site^25,26^. Tissue biopsy of the anterior orbit is feasible without complications, particularly in patients without a history of cancer. In contrast, deep orbital lesions and choroidal tumors are the leading causes of optic nerve or retinal injury; thereby contributing to a challenging biopsy.

Survival rates depend on the origin of the primary tumor; however, OS remains poor. A quarter of all patients refused treatment for their stage 4 cancer for reasons including severe sickness at presentation, preference for observation or herbal medicine, and lack of targeted therapy. Patients with liver cancer have a worse survival, whereas those with breast cancer have the longest survival^27^. Despite poor survival, the patients who underwent treatment for primary cancer and metastasis control tended to gain VA, which affected their quality of life in late-stage disease. Targeted therapy and immunotherapy improve the long-term survival rates^28^.

The study has some limitations, which include its retrospective design, small sample size, missing data, and selection bias. The relatively small number of 42 patients restricted the ability to determine significant differences between the subgroups. Most patients in the IM group were diagnosed based on clinical features and investigations, without biopsy-proven diagnoses, owing to the limited benefit of choroidal mass biopsy in severely ill patients, unlike in the indirect evidence of primary tumor origin and other metastatic sites. By contrast, the OAM group included patients predominantly diagnosed using histopathologic reports. The exact incidences of IM and OAM may be underreported because of severe clinical illness, asymptomatic cases of advanced cancer, and inadequate histopathological results or comprehensive investigations. However, the rarity of this disease hinders prospective studies. This limitation warrants a multicenter study with a large sample size and additional data acquired during the follow-up period.

In conclusion, this novel cohort study reported on the primary tumor sites, OS, and visual outcomes of IM and OAM in Southeast Asians. Ophthalmologists are central to the diagnosis of this rare but fatal condition. They conduct extensive investigations to identify the primary tumor origin because the patients commonly present with ocular symptoms without a history of cancer. Lung cancer commonly metastasizes to the uvea, and breast cancer affects the orbit, which should be the target organ of investigation. Despite poor survival rates, treatment can improve the OS and vision.

## Data Availability

All unidentifiable data are available at the corresponding author upon reasonable request.
