# Author Correction: Clinical features and treatment outcomes of intraocular and ocular adnexal metastasis

**DOI:** 10.1038/s41598-024-69894-3

**Published:** 2024-08-13

**Authors:** Wantanee Dangboon Tsutsumi, Apinya Rattanasuwan, Orapan Aryasit

**Affiliations:** Department of Ophthalmology, Faculty of Medicine, Prince of Songkla University, 15, Kanjanavanich Road, Kohong, Hat Yai, 90110 Songkhla Thailand

Correction to: *Scientific Reports* 10.1038/s41598-024-64464-z, published online 03 July 2024

The original version of this Article contained errors in the Figure legends of Figure 1 and Figure 2. The legends of these Figures were inadvertently switched.

The legend of Figure 1:

“Kaplan–Meier survival estimates of the overall survival. (**a**) All patients; (**b**) age ≤ 50 years vs. > 50 years; (**c**) primary cancer origin; and (**d**) treatment group vs. observation group.”

now reads:

“A 26-year-old woman presents with flashing and reduced vision in the right eye: (**a**) Color fundus photography indicates pale yellow choroidal mass; (**b**) Hypo-reflective elevated choroidal mass with subretinal fluid on optical coherence tomography; (**c**) Ultrasonography shows a hypoechoic dome shape mass with moderate variable amplitude of internal reflectivity on A-scan; and (**d**) Lung mass with poorly differentiated adenocarcinoma.”

The legend of Figure 2:

“A 26-year-old woman presents with flashing and reduced vision in the right eye: (**a**) Color fundus photography indicates pale yellow choroidal mass; (**b**) Hypo-reflective elevated choroidal mass with subretinal fluid on optical coherence tomography; (**c**) Ultrasonography shows a hypoechoic dome shape mass with moderate variable amplitude of internal reflectivity on A-scan; and (**d**) Lung mass with poorly differentiated adenocarcinoma.”

now reads:

“Kaplan–Meier survival estimates of the overall survival. (**a**) All patients; (**b**) age ≤ 50 years vs. > 50 years; (**c**) primary cancer origin; and (**d**) treatment group vs. observation group.”

The original Article has been corrected.

